# Effects of safinamide on non-motor, cognitive, and behavioral symptoms in fluctuating Parkinson’s disease patients: a prospective longitudinal study

**DOI:** 10.1007/s10072-021-05324-w

**Published:** 2021-05-24

**Authors:** Rosa De Micco, Sara Satolli, Mattia Siciliano, Antonio De Mase, Alfonso Giordano, Gioacchino Tedeschi, Alessandro Tessitore

**Affiliations:** 1 Department of Advanced Medical and Surgical Sciences - MRI Research Center Vanvitelli-FISM, University of Campania “Luigi Vanvitelli”, Piazza Miraglia 2, 80138 Naples, Italy; 2 Department of Psychology, University of Campania “Luigi Vanvitelli”, Viale Ellittico 31, 81100 Caserta, Italy

**Keywords:** Parkinson’s disease, Safinamide, Fluctuations, Behavioral symptoms, Urinary dysfunction

## Abstract

**Introduction:**

Parkinson’s disease (PD) patients in chronic levodopa treatment may experience motor and non-motor fluctuations, which may affect their quality of life. Safinamide is a new monoamine oxidase B inhibitor, also exerting a non-dopaminergic effect, recently approved as add-on therapy in fluctuating PD patients.

**Methods:**

We performed a longitudinal prospective study in a cohort of 20 fluctuating PD patients, to test whether safinamide 50 mg may improve non-motor, cognitive, and behavioral symptoms over a 6-month treatment period. At each timepoint, clinical features were assessed by means of validated PD-specific scales. Neuropsychological assessment was performed by exploring all five cognitive domains.

**Results:**

Compared to baseline, significant improvement was found in PD patients at 6-month follow-up in items investigating interest (p = 0.02), motivation (p = 0.02), and urinary disturbances (p = 0.03). Moreover, neuropsychiatric assessment showed a significant decrease in fatigue and apathy scores (p = 0.02 and p = 0.01, respectively). Motor assessment revealed a significant reduction in the total wake-up time spent in OFF state (p = 0.01). Follow-up neuropsychological evaluation did not reveal any change compared to baseline.

**Conclusions:**

Our data reveal that, along with motor fluctuation improvement, treatment with safinamide 50 mg may significantly decrease non-motor symptom burden in PD patients. Interestingly, non-dopaminergic mechanisms, such as glutamatergic overdrive, have been demonstrated to play a role in many pathways underlying these symptoms. Thus, we hypothesize that the neurotransmitter receptor-binding profile of safinamide may explain our findings.

**Supplementary Information:**

The online version contains supplementary material available at 10.1007/s10072-021-05324-w.

## Introduction

Parkinson’s disease (PD) is a common and complex neurodegenerative disorder characterized by motor features, such as slowness of movement, rigidity, and resting tremor as well as several and heterogenous non-motor symptoms (i.e., cognitive impairment, psychiatric symptoms, sleep disorders, autonomic dysfunction, pain, and fatigue) [1].

Despite their clinical relevance, PD- related non-motor symptoms are still poorly recognized and inadequately treated [2]. This may be related to the fact that their neuroanatomical correlates in PD remain largely undefined, with limited insights for treatment options. It has been hypothesized that a dysfunction of both dopaminergic and non-dopaminergic systems may contribute to their development over the disease course [3].

Levodopa is the most effective symptomatic treatment for motor features in PD. Complications of long-term dopaminergic treatment are motor and non-motor fluctuations and dyskinesia, which can impair patients’ quality of life [4].

Fragmentation of levodopa doses and combination with catechol-O-methyl transferase (COMT) and monoamine oxidase B inhibitors (iMAO-B) or dopamine agonists (DA) are the most common pharmacological strategies to treat motor fluctuations [5].

Safinamide has been approved as an add-on therapy to levodopa in mid-stage to late-stage fluctuating PD patients [6]. Several large phase III clinical trials have shown that safinamide, administered orally at doses of 50 or 100 mg daily in patients with PD and motor fluctuations, increased ON time with no or non-troublesome dyskinesia and decreased daily OFF time [6]. Preclinical and human studies have shown that safinamide presents both dopaminergic and non-dopaminergic (i.e., glutamatergic) properties [7, 8]. Indeed, acting as a highly selective iMAO-B, safinamide reduces dopamine re-uptake in the synaptic junction, increasing dopamine levels. This effect is fully achieved at the lowest dose of 50 mg [7, 9]. Moreover, safinamide has a non-dopaminergic mechanism of action, by blocking voltage-gated sodium channels and N-type calcium channels, modulating glutamate release, and causing NMDA receptor-antagonizing effects [7–9].

Although the effect of safinamide on abnormal glutamate release may be optimal at a dose of 100 mg/day [10], safinamide also acts on the same circuits at a dose of 50 mg/day. It is important to note that the antiglutamatergic effect of the two recommended doses of safinamide has shown a clinical relevance in patients with refractory partial and/or generalized epilepsy [9]. This effect has been recently further supported by the modulatory effect exerted by safinamide at both 50 and 100 daily dose on the abnormal cortical facilitatory circuits in PD patients with levodopa-induced dyskinesia [11].

Based on its peculiar mechanism of action, it has been suggested that safinamide may improve non-motor symptoms. However, this hypothesis has not been yet adequately verified. Indeed, most trials on safinamide have used motor features as their primary outcome measures and included non-motor assessment tools only as secondary outcome measures [12]. Overall, these studies showed that treatment with safinamide is associated with better quality of life as well as with improvement of non-motor symptoms such as pain, mood, and sleep [13–17]. To our knowledge, there is no longitudinal study specifically designed to focus the prospective effect of safinamide on a large spectrum of non-motor, cognitive, and behavioral PD-related features. Thus, the aim of the present study is to investigate, by means of several PD-specific and validated scales, whether safinamide may improve these symptoms over a 6-month treatment period in a cohort of mid-stage PD patients experiencing motor fluctuations.

## Materials and methods

### Study population

Patients were consecutive cases clinically diagnosed with idiopathic PD according to the diagnostic criteria of the UK Parkinson’s Disease Society Brain Bank [e1] attending the Movement Disorders Unit of the First Division of Neurology at the University of Campania “Luigi Vanvitelli” (Naples, Italy) from January 2018 to April 2018. Inclusion criteria were (a) PD onset after the age of 40 years to exclude early-onset parkinsonism; (b) presence of motor fluctuations (>1.5 h OFF time/day); and (c) stable dopaminergic treatment in the last 30 days. Exclusion criteria were (a) previous treatment with safinamide; (b) dementia associated with PD according to consensus criteria [e2, e3]; (c) major depression and/or dysthymic disorder according to DSM-IV criteria and/or history of or current psychiatric illness (i.e., hypomanic or manic episodes, psychosis, substance abuse, and attention-deficit hyperactivity disorder); (d) any other neurological disorder or clinically significant or unstable medical condition; and (e) any contraindications to the treatment with safinamide.

We obtained written informed consent from all subjects. The study was approved by the ethics committee of the University of Campania “Luigi Vanvitelli,” Naples, Italy.

### Study design

Patients underwent an extensive clinical evaluation to assess motor, non-motor, cognitive, and behavioral features by means of international validated scales (see below). Clinical data were collected at baseline, in the morning, in distinct sessions. Short breaks were introduced to avoid fatigue.

After the baseline assessments, all patients added safinamide 50 mg daily to their dopaminergic treatment. Patients already taking other iMAO-B, such as rasagiline or selegiline, were switched to safinamide 50 mg after a 15-day wash-out period. After 6 months of treatment, clinical features were re-assessed by the same clinicians and treatment details were registered.

### Motor and non-motor evaluation

At baseline and at the 6-month follow-up visit, motor symptom severity was assessed using the modified Hoehn and Yahr (mH&Y) stage [e4] and the Unified Parkinson’s Disease Rating Scale part III (UPDRS III) [e5] in ON state. Moreover, the presence of treatment-related motor complications was assessed by means of the UPDRS part IV [e5]. In details, items 32-33 and 39 were used to allow for calculation of hours of ON time with dyskinesia, dyskinesia severity, and actual hours of OFF time, respectively. Moreover, the Wearing-off questionnaire 19 (WOQ-19) [e6] and the Abnormal Involuntary Movements Scale (AIMS) [e7] were also used to determine the presence and severity of treatment complications. At each visit, the levodopa equivalent daily dose (LEDD) was calculated for both dopamine agonists (LEDD-DA) and dopamine agonists + L-dopa (total LEDD) [e8].

Non-motor symptoms were assessed using the Non-Motor Symptom Scale (NMSS) [e9]. Patient-reported quality of life (QoL) was evaluated using the Parkinson's Disease Questionnaire 39 (PDQ-39) [e10].

### Neuropsychological and behavioral assessments

At baseline and at follow-up, neuropsychological and behavioral assessments were performed in the ON condition. Global cognitive functioning was assessed by means of the Montreal Cognitive Assessment (MoCA [e11]; score range: 0-30 points), and the PD Cognitive Rating Scale (PD-CRS [e12, e13]; score range: 0-134 points). Two subscores were derived from PD-CRS, assessing “frontal-subcortical” functions (score range: 0-104 points), and “instrumental-cortical” functions (score range: 0-30 points).

Depressive symptoms were assessed by means of the Beck Depression Index (BDI) [e14]. Anxiety was assessed with the Parkinson Anxiety Scale (PAS) [e15]. Autonomic dysfunction was assessed with the Scales for Outcomes in Parkinson’s Disease–Autonomic (SCOPA-AUT) [e16]. Sleep disturbances were assessed with the Epworth Sleepiness Scale (ESS) [e17] and the PD Sleep Scale (PDSS-2) [e18]. The Questionnaire for Impulsive Compulsive Disorders in PD rating scale (QUIP-RS) was used to assess the presence and severity of impulse control disorders and other compulsive behaviors (i.e., punding, hobbyism, and walkabout) [e19]. King’s PD Pain (KPP) scale was used to assess the presence and severity of pain symptoms [e20]. Apathy symptoms were assessed with the Apathy Evaluation Scale (AES) [e21]. Fatigue was assessed with the Parkinson’s disease Fatigue Scale (PFS) [e22].

### Statistical analysis

Within-subject longitudinal differences on demographic and clinical variables between the two timepoints were assessed by means of one-way repeated-measures ANOVA analyses. Longitudinal changes of each clinical variable (*i*) were calculated as follows: ∆*i =* (score *i*_follow-up_ − mean score *i*_baseline_)/standard deviation score *i*_baseline_. For motor, non-motor, and behavioral scores, greater ∆*i* indicates less improvement of symptoms over time, while lower ∆*i* indicates higher worsening of cognitive scores over time. Pearson correlations were used to test the relationship between longitudinal changes of non-motor, cognitive, and behavioral variables and motor variables (i.e., mH&Y, UPDRS III, UPDRS IV) after 6 months of treatment with safinamide.

A p < 0.05 was considered statistically significant. Analyses were performed with SPSS version 13 (SPSS Inc. Chicago, IL).

## Results

### Motor and treatment features

After the baseline assessment, 20 fluctuating PD patients were enrolled in the study and after add-on with safinamide 50 mg performed a 6-month extensive follow-up. Eleven out of 20 PD patients were taking rasagiline as iMAO-B prior to the inclusion in this study; therefore, they were switched on safinamide. Demographic and clinical characteristics of PD patients at baseline are summarized in Table 1. Seven out of 20 PD patients were presenting with dyskinesia combined with other motor fluctuations (i.e., wearing-off). Mostly, PD patients were taking more than one antiparkinsonian drugs, mainly COMT inhibitors and DAs. At both the baseline and the follow-up visits, no patients were taking antidepressants and/or anxiolytics and/or anticholinergics and/or anticonvulsants. Eight patients suffered from hypertension, 4 patients suffered from diabetes (type 2, without neurological complications), 5 patients were dyslipidemic, 2 patients were affected by benign prostatic hyperplasia, and 1 patient by hypothyroidism.

**Table 1:** Clinical and demographic features at baseline

Variables	PD patients (n = 20) mean ± SD/count (%)
Age (y)	63.8 ± 10.24
Male sex	11 (55%)
mH&Y stage	2.22 ± 0.41
Disease duration (y)	6 ± 2.22
UPDRS III	24.41 ± 8.9
Total LEDD	640.75 ± 223.44
LEDD levodopa	525.00 ± 190.91
LEDD-DA	65.75 ± 112.01
UPDRS IV	3.8 ± 2.57
AIMS	0.9± 2.31
MoCA (tot)	21.98 ± 3.27
Fluctuations	
Dyskinesia n (%)	7 (35%)
Wearing-off n (%)	20 (100%)
Both n (%)	7 (35%)
Duration of fluctuations (m)	16.5 ± 14.6

*PD* Parkinson’s disease; *H&Y* Hoehn & Yahr; *UPDRS* Unified Parkinson’s Disease Rating Scale; *AIMS* Abnormal Involuntary Movements Scale; *MoCA* Montreal Cognitive Assessment; *LEDD* levodopa equivalent daily dose; *DA* dopamine agonist; *y* years; *m* months

According to the UPDRS III, 17 PD patients were classified as akinetic-rigid subtype and 3 patients were classified as tremor-dominant subtype.

Patients’ characteristics at follow-up are listed in Tables 2 and 3. Total daily hours spent in the OFF state (UPDRS IV item 39) significantly decreased from a value of 1.6 ± 0.82 (mean ± SD) at baseline to 1 ± 0.75 (mean ± SD) at follow-up (p = 0.01, Table 2). No significant changes were detected in terms of total daily time spent with dyskinesia as well as dyskinesia severity (UPDRS IV item 32-33, AIMS) after 6 months of treatment with safinamide.

**Table 2:** Longitudinal clinical motor and treatment features after 6 months of treatment with safinamide

Variables	PD patient pre-treatment mean ± SD/count (%)	PD patient post-treatment mean ± SD/count (%)	p
	(n = 20)	(n = 20)	
UPDRS III	24.41 ± 8.9	27.35 ± 10.13	0.34
UPDRS IV	4.3 ± 2.15	3.8 ± 2.16	0.47
UPDRS IV (item 32. Dyskinesias wake-up time)	0.35 ± 0.28	0.5 ± 1.1	0.65
UPDRS IV (item 33. Disabling dyskinesias wake-up time)	0.11 ± 0.30	0.05 ± 0.22	0.54
UPDRS IV (item 39. OFF wake-up time)	1.6 ± 0.82	1 ± 0.75	**0.01**
AIMS	0.90 ± 2.31	1.55 ± 2.96	0.44
Total LEDD	640.75 ± 223.44	584.75 ± 160.93	0.37
LEDD levodopa	525.00 ± 190.91	540.75 ± 142.63	0.77
LEDD-DA	65.75 ± 112.01	44.00 ± 79.89	0.48
Fluctuations			
Dyskinesia n (%)	7 (35%)	10 (50%)	0.92
Wearing-off n (%)	20 (100%)	16 (80%)	0.07

Significant differences are reported in bold

*PD* Parkinson’s disease; *UPDRS* Unified Parkinson’s Disease Rating Scale; *AIMS* Abnormal Involuntary Movements Scale; *LEDD* levodopa equivalent daily dose; *DA* dopamine agonist

**Table 3:** Longitudinal clinical non-motor features after 6 months of treatment with safinamide

Variables	PD patient pre-treatment mean ± SD	PD patient post-treatment mean ± SD	p
	(n = 20)	(n = 20)	
PDQ39	43.11 ± 28.80	33.40 ± 23.64	0.25
BDI	6.9 ± 5.05	6.7 ± 5.93	0.91
PAS	12.00 ± 8.62	10.65 ± 6.87	0.59
KPP	9.40 ± 7.88	8.60 ± 9.20	0.77
QUIP-RS	0.75 ± 2.04	1.15 ± 2.18	0.55
SCOPA-AUT	12.8 ± 5.69	7.95 ± 4.40	**0.04**
ESS	5.05 ± 3.55	4.20 ± 2.97	0.42
PDSS-2	117.2 ± 21.44	121.4 ± 17.66	0.50
NMSS (tot)	57.8 ± 50.72	33.45 ± 24.2	**0.02**
NMSS 3.7 (interest)	2.85 ± 4.25	0.8 ± 1.85	**0.02**
NMSS 3.8 (motivation)	3.55 ± 4.54	0.75 ± 1.74	**0.02**
NMSS 7.23 (urine frequency)	3.85 ± 4.85	0.55 ± 1.43	**0.03**
PFS	2.85 ± 0.67	2.20 ± 1.07	**0.02**
AES	34.65 ± 7.41	30.35 ± 7.8	**0.01**

Significant differences are reported in bold

*PD* Parkinson’s disease; *PDQ-39* PD Questionnaire 39; *BDI* Beck Depression Inventory; *PAS* PD Anxiety Scale; *KPP* King’s PD Pain Scale; *QUIP-RS* Questionnaire for Impulsive Compulsive Disorders in PD rating scale; *SCOPA-AUT* Scales for Outcomes in Parkinson’s Disease–Autonomic; *ESS* Epworth Sleepiness Scale; *PDSS-2* PD Sleep Scale version 2; *NMSS* Non-Motor Symptoms Scale; *PFS* PD Fatigue Scale; *AES* Apathy Evaluation Scale

### Non-motor features

Compared to baseline, NMSS total scores significantly decreased at follow-up (p = 0.02). Specifically, a significant improvement was observed in 3 out of the 30 NMSS sub-domains: domain 3.7 (interest, p = 0.02), domain 3.8 (motivation, p = 0.02), and domain 7.23 (urine frequency, p = 0.03) (Table 3).

Furthermore, SCOPA-AUT total score significantly decreased at the follow-up visit compared to baseline (p = 0.04) (Table 3).

### Behavioral features

PFS and AES significantly improved at follow-up visit compared to baseline (p = 0.02 and p = 0.01, respectively) (Table 3).

### Cognitive features

Neuropsychological evaluations did not reveal any change at follow-up compared to baseline in both global cognitive functioning and any of the five cognitive sub-domains (Table 4).

**Table 4:** Longitudinal clinical cognitive features after 6 months of treatment with safinamide

Variables	PD patient pre-treatment mean ± SD	PD patient post-treatment mean ± SD	p
	(n = 20)	(n = 20)	
MoCA (tot)	21.98 ± 3.27	21.90 ± 3.6	0.94
PD-CRS (tot)	83.77 ± 12.54	87.99 ± 14.39	0.37
PD-CRS (subcortical)	57.90 ± 11.39	62.13 ± 13.16	0.40
PD-CRS (cortical)	25.38 ± 2.82	25.54 ± 3.83	0.87

*PD* Parkinson’s disease; *PD-CRS* PD Cognitive Rating Scale; *MoCA* Montreal Cognitive Assessment

### Correlation analysis

A positive correlation was found between higher mH&Y scores at baseline and ∆PAS (i.e., less improvement of anxiety symptoms over time, p = 0.005, R = 0.60) and ∆ESS (i.e., less improvement of sleepiness symptoms over time, p = 0.01, R = 0.56). A negative correlation was found between higher mH&Y scores at baseline and ∆PD-CRS (i.e., less improvement of cognitive symptoms over time, p = 0.02, R = −0.51). A positive correlation was found between higher ∆UPDRS III scores (i.e., less improvement in motor outcome over time) and ∆BDI (i.e., less improvement of depressive symptoms over time, p = 0.04, R = 0.46), ∆PAS (i.e., less improvement of anxiety symptoms over time, p = 0.03, R = 0.48), ∆PFS (i.e., less improvement of fatigue symptoms over time, p = 0.007, R = 0.58), and ∆AES (i.e., less improvement of apathy symptoms over time, p = 0.03, R = 0.48). No other correlations were found between motor and non-motor and cognitive and behavioral outcomes after 6 months of treatment with safinamide.

### Adverse events

Adverse events are reported in Supplementary Table 1. Safinamide at the dose of 50 mg/daily was safe and well tolerated, and no major or unexpected safety concerns were identified.

## Discussion

In this prospective longitudinal study, we extensively tested the effects of safinamide on non-motor, cognitive, and behavioral symptoms in PD patients with motor fluctuations.

Our findings confirmed that safinamide as add-on therapy in fluctuating PD patients can significantly reduce the daily time spent in OFF condition and increase daily ON time without troublesome dyskinesia. This is consistent with previous studies with longer follow-up [12].

Most importantly, our findings demonstrate that long-term treatment with safinamide 50 mg may effectively improve interest, motivation, apathy, fatigue, and urinary symptoms over 6 months, while its effect on cognitive symptoms was unremarkable.

In details, PD patients reported a significant improvement in specific NMSS items regarding interest and motivation, which it is likely to parallel the statistically significant decreasing in apathy severity (i.e., AES scores).

Several studies have provided strong evidence that dopaminergic pathways may contribute to the pathophysiology and treatment of mood disorders. Evidence from neuroimaging studies have shown a decreased meso-cortico-limbic dopaminergic activity in non-depressive apathetic PD patients [18]. Both the presence and severity of isolated apathy were related to gray matter volume loss in dopaminergic-driven areas such as the nucleus accumbens, the anterior cingulate cortex, and the medial prefrontal cortex [19]. Apathy severity was also related to neurofibrillary tangles density in the anterior cingulate gyrus, while a decreased dopamine binding and metabolism has been observed in the ventral striatum of apathetic PD patients [18].

In vivo animal and human studies have demonstrated that these areas may play an important role in reward-guided behaviors. Taking into account these findings, the dopaminergic targeting of safinamide 50 mg may underpin the effect we found on interest, motivation, and apathy after 6 months of treatment. This hypothesis is further corroborated by our correlation analysis, which showed that greater improvement in apathy symptoms was correlated with greater improvement in motor outcome after safinamide add-on.

In addition to dopamine, other neurotransmitters have been implicated in behavioral driving.

Indeed, in vivo studies have demonstrated that NMDA receptor blockade in the medial prefrontal cortex strongly potentiates opiate reward processing and also modulates subcortical dopaminergic firing within the ventral tegmental area [20].

As already discussed, these pathways have been shown to be targeted from safinamide even at the lowest dose [10] further supporting our hypothesis of a multimodal effect of safinamide on apathy symptoms, with both an increase of dopamine availability and dopaminergic firing (via NMDA blocking) in the meso-cortico-limbic-circuit.

We have also observed an effect of safinamide on fatigue severity (i.e., PFS scores).

To date, the pathophysiology of fatigue in PD remains still poorly understood. Several studies suggested that fatigue in PD is not strictly related to mood disorders [21]. The improvement of fatigue severity has been shown to parallel a reduction in OFF periods [22], but a clear association with type, duration, or dosage of dopaminergic medication has been not yet demonstrated [21]. Our recent meta-analysis did not find any association between fatigue and motor symptoms, supporting the hypothesis that dopaminergic dysfunction alone may be not sufficient to determine the development of fatigue [23].

Other mechanisms have been proposed to underlie fatigue in PD, such as the involvement of non-dopaminergic (mainly serotonergic) pathways, as well as increased levels of circulating proinflammatory cytokines, greater executive/prefrontal pathology, and dysautonomic involvement [24]. Similarly to apathy symptoms, patients with fatigue (with and without PD) showed reduced glucose metabolism in the prefrontal cortex, and this potentially supports the hypothesis that NMDA-related dysfunction within the cortico-striatal reward circuitry may, at least partially, underlie fatigue [25]. However, we acknowledge that at this time this hypothesis is merely speculative, as no evidence of glutamatergic involvement in the pathophysiology of fatigue has been found in PD patients.

We also found a possible effect of safinamide on urinary symptoms, as PD patients showed decreased severity of urinary frequency at 6-month follow-up compared to baseline. Several studies demonstrated that urinary symptoms in PD may improve with dopaminergic therapy [26]. Interestingly, our data are consistent with a recent study showing improvement of urinary function in PD patients treated with safinamide 100 mg [27]. Although voiding is regulated by the peripheral autonomic nervous system, it can be influenced by facilitatory and inhibitory impulses from higher cortical centers, such as the dopaminergic basal ganglia circuit. Specifically, it has been demonstrated that dopaminergic D1 receptors are inhibitory, whereas D2 stimulation facilitates micturition. It has been proposed that urinary dysfunction in PD may be related to the loss of this D1-mediated inhibition, leading to detrusor overactivity [28]. Moreover, urodynamic studies have confirmed a higher prevalence of detrusor overactivity in PD [29, 30]. Interestingly, glutamatergic neurotransmission may also affect the micturition reflex. Glutamate injection into the pontine micturition center can trigger a micturition reflex in animals, indicating glutamatergic excitatory mechanisms [31]. Furthermore, bladder hyperactivity has been shown to be linked to the upregulation of NMDA receptors in animals [32]. Thus, both glutamatergic and dopaminergic effects of safinamide could potentially explain our results of urinary function improvement after 6 months of treatment.

We did not find significant effects of safinamide treatment on pain symptoms. This is apparently in contrast with recent studies [13, 16, 33], showing significant improvement in pain symptom severity following safinamide add-on [16]. Some of these studies [13, 16] were post hoc analyses; thereby, they were not specifically designed to investigate pain as a primary endpoint. Indeed, pain presence and severity were rated by means of PDQ39 subitems (i.e., “bodily discomfort”) and reduction in analgesic use was also evaluated as indirect measure of pain improvement [13, 16].

Moreover, in these studies [13, 16, 33], the effects of safinamide on pain symptoms were tested at the target dose of 100 mg/day. It is well known that abnormalities in pain processing may result from dysfunction of both dopaminergic and non-dopaminergic pathways, including glutamatergic [34, 35]. Although safinamide acts on the same circuits at a dose of 50 mg/day, the effect on abnormal glutamate release may be optimal at a dose of 100 mg/day [10]. This may partially explain the lack of effect on pain symptoms in the present study at the dose of 50 mg.

Another possible divergence between our studies and previous works may be the PD sample [33]. For example, Geroin et al. recently performed a prospective study on PD patients with motor fluctuation and chronic specifically focusing those patients complaining moderate to severe pain symptoms and found an effect of safinamide on pain over 12 weeks of treatment [33].

Finally, our PD sample showed stable cognitive functioning over 6 months of treatment with safinamide.

Beyond the nigrostriatal pathway, spreading of the neurodegeneration over dopaminergic neurons in the ventro-medial caudate as well as in the meso-cortico-limbic pathway has been associated with the development of cognitive impairment in PD [36]. From these structures, brain regions which are crucial for executive functions, such as the prefrontal cortex, become progressively involved [36]. Safinamide may potentially modulate the dopamine availability within these pathways [37]. This hypothesis is supported by a recent study, which showed that safinamide add-on may improve executive functions in patients with PD [38].

Interestingly, pre-human studies have suggested that glutamatergic NMDA-mediated neurotransmission may be involved in the pathophysiology of Alzheimer’s disease, also modulating the formation of amyloid and tau protein aggregates [39]. Together these findings may potentially explain the presence of a stable cognitive functioning we found in this study over 6 months of treatment with safinamide.

On the other hand, previous studies showed that cognitive annual decline rate of MoCA scores in patients with PD is only 1.02 points [40]. MoCA is a well-validated screening instrument that evaluates different cognitive domains and scores range from 0 to 30 with a detectable score difference of 1 point. Thus, this tool may be not sufficiently sensitive to display minimal score differences in a short follow-up period. We acknowledge that a longer follow-up period would have been necessary to potentially detect any improvement in specific cognitive sub-domains.

In conclusion, the consistent significant and clinically relevant improvement over 6 months in motor outcome confirms the effectiveness of safinamide 50 mg in fluctuating patients with PD. Moreover, we have demonstrated that non-motor and behavioral PD-related symptoms may benefit from safinamide treatment, even at the lowest available dose of 50 mg. The neurotransmitter receptor-binding profile of safinamide, which has been shown to modulate dopaminergic and also glutamatergic transmission, may potentially explain this effect. Future studies with longer follow-up periods and a larger number of patients are needed to confirm and possibly expand our results.

## Supplementary information

ESM 1 (DOC 28 kb).

ESM 2 (DOCX 56 kb).

ESM 3 (DOCX 26 kb).

ESM 4 (DOC 52 kb).
