# Supplementary material for: Effects of safinamide on non-motor, cognitive, and behavioral symptoms in fluctuating Parkinson’s disease patients: a prospective longitudinal study
Source: Neurol Sci. 2021 May 24;43(1):357–64. doi: 10.1007/s10072-021-05324-w (PMC8724100; doi:10.1007/s10072-021-05324-w)
Supplement: Supplementary file 1 — (DOC 28 kb). [file 10072_2021_5324_MOESM1_ESM.doc]

**Supplementary table 1. Adverse events reported during safinamide treatment period**

| **Event** | **Number of patients (%)**  **(n=20)** |
| --- | --- |
| Nausea | 1 (5%) |
| Dizziness | 1 (5%) |
| Dysgeusia | 1 (5%) |
| Dyskinesia | 1 (5%) |
