# Supplementary material for: Effects of safinamide on non-motor, cognitive, and behavioral symptoms in fluctuating Parkinson’s disease patients: a prospective longitudinal study
Source: Neurol Sci. 2021 May 24;43(1):357–64. doi: 10.1007/s10072-021-05324-w (PMC8724100; doi:10.1007/s10072-021-05324-w)
Supplement: Supplementary file 2 — (DOCX 56 kb). [file 10072_2021_5324_MOESM2_ESM.docx]

**Table S2.** Baseline and follow-up clinical features of PD patients with different motor phenotypes

| **Variables** | **Akinetic-rigid PD** | | **Tremor-dominant PD** | | ***P***  **Akinetic-rigid**  **vs**  **Tremor-dominant**  **at baseline** | ***P***  **Akinetic-rigid**  **vs**  **Tremor-dominant**  **at follow-up** | ***P***  **for linear trend**  **Akinetic-rigid** | ***P***  **for linear trend**  **Tremor-dominant** | ***P***  **Akinetic-rigid**  **vs**  **Tremor-dominant**  **Longitudinal changes** |
| --- | --- | --- | --- | --- | --- | --- | --- | --- | --- |
|  | **Baseline** | **6-month**  **follow-up** | **Baseline** | **6-month**  **follow-up** |  |  |  |  |  |
| **N** | **17** | | **3** | |  |  |  |  |  |
| **Age (years)** | 63.2±10.1 | - | 67.0±12.5 | - | N.S. | - | - | - | - |
| **Gender (M/W)** | 10/7 | - | 1/2 | - | N.S. | - | - | - | - |
| **UPDRS III** | 24.4±9.6 | 27.8±10.2 | 24.7±4.7 | 25.0±11.3 | N.S. | N.S. | N.S. | N.S. | N.S. |
| **Total LEDD (mg)** | 671.5±228.4 | 599.7±168.0 | 466.7±57.7 | 500.0±86.7 | 0.007 | N.S. | N.S. | N.S. | N.S. |
| **LEDD Dopa (mg)** | 541.2±202.5 | 547.9±151.2 | 433.3±57.7 | 500.0±86.7 | N.S. | N.S. | N.S. | N.S. | N.S. |
| **LEDD DA (mg)** | 77.3±118.1 | 51.7±84.6 | 0.0±0.0 | 0.0±0.0 | 0.016 | 0.023 | N.S. | N.S. | 0.023 |
| **UPDRS IV** | 4.0±2.7 | 4.2±2.0 | 2.7±1.5 | 1.7±1.1 | N.S. | N.S. | N.S. | N.S. | N.S. |
| **AIMS** | 1.0±2.5 | 1.7±3.2 | 0.0±0.0 | 0.3±0.6 | N.S. | N.S. | N.S. | N.S. | N.S. |
| **NMSS total** | 54.7±51.9 | 35.5±25.5 | 75.3±48.8 | 22.0±12.0 | N.S. | N.S. | N.S. | N.S. | N.S. |
| **NMSS Item 1.1** | 0.8±1.5 | 0.5±1.2 | 0.0±0.0 | 0.0±0.0 | 0.027 | N.S. | N.S. | N.S. | N.S. |
| **NMSS Item 3.7** | 2.0±3.6 | 0.9±1.9 | 7.7±5.1 | 0.0±0.0 | 0.029 | N.S. | 0.012 | 0.024 | N.S. |
| **NMSS Item 5.16** | 2.6±3.7 | 0.8±1.6 | 0.0±0.0 | 3.0±5.2 | 0.010 | N.S. | 0.05 | N.S. | N.S. |
| **NMSS Item 5.18** | 1.7±3.9 | 0.8±1.5 | 0.0±0.0 | 0.0±0.0 | N.S. | N.S. | N.S. | N.S. | 0.039 |
| **NMSS Item 6.21** | 2.3±3.3 | 2.2±3.8 | 8.7±5.7 | 1.7±2.0 | 0.012 | N.S. | N.S. | N.S. | N.S. |
| **NMSS Item 7.22** | 3.4±4.1 | 3.0±3.2 | 7.0±6.2 | 0.0±0.0 | N.S. | 0.001 | N.S. | N.S. | 0.001 |
| **NMSS Item 7.26** | 1.2±3.5 | 0.0±0.0 | 0.0±0.0 | 0.0±0.0 | N.S. | N.S. | N.S. | N.S. | <0.001 |
| **BDI** | 6.6±5.2 | 7.0±6.0 | 8.7±3.5 | 4.7±5.7 | N.S. | N.S. | N.S. | N.S. | N.S. |
| **PAS** | 11.5±8.7 | 7.0±4.5 | 15.0±8.6 | 6.7±3.0 | N.S. | N.S. | N.S. | N.S. | N.S. |
| **KPP** | 8.0±6.8 | 8.8±9.8 | 17.3±10.0 | 7.3±5.0 | N.S. | N.S. | N.S. | N.S. | N.S. |
| **QUIP-RS** | 0.6±1.5 | 1.0±1.8 | 0.0±0.0 | 0.0±0.0 | N.S. | 0.029 | N.S. | N.S. | 0.029 |
| **ESS** | 5.2±3.7 | 4.0±3.1 | 4.0±2.0 | 3.3±1.1 | N.S. | N.S. | N.S. | N.S. | N.S. |
| **PDSS-2** | 117.1±23.3 | 121.6±18.5 | 117.7±4.3 | 120.3±150 | N.S. | N.S. | N.S. | N.S. | N.S. |
| **PFS** | 2.6±1.0 | 2.7±1.3 | 3.3±0.7 | 1.2±0.7 | N.S. | N.S. | N.S. | 0.031 | 0.026 |
| **AES** | 34.2±7.7 | 29.8±8.0 | 37.0±6.0 | 33.3±7.6 | N.S. | N.S. | N.S. | N.S. | N.S. |
| **MoCA** | 21.9±3.5 | 21.3±3.4 | 22.4±2.3 | 25.4±3.0 | N.S. | N.S. | N.S. | N.S. | N.S. |
| **PD-CRS (tot)** | 84.5±12.3 | 88.1±14.5 | 77.7±17.4 | 87.2±16.5 | N.S. | N.S. | N.S. | N.S. | N.S. |

Values are reported as mean ± standard deviation. Baseline and follow-up scores were compared by means of *t*-tests or Chi-squared tests as appropriate. For linear trend analysis, *P* values refer to repeated measures ANOVA models. One-way ANOVA was used to explore the differences in longitudinal changes of each clinical variable [(score *i*_follow-up_ – mean score *i*_baseline_) / Standard Deviation score *i*_baseline_] between the two PD subgroups. Abbreviations: N.S.: not significant; PD: Parkinson’s disease; UPDRS: Unified Parkinson’s Disease Rating Scale: AIMS: Abnormal Involuntary Movements Scale; LEDD: Levodopa Equivalent Daily Dose; DA: dopamine agonist; BDI: Beck Depression Inventory; PAS: PD Anxiety Scale; KPP: King’s PD Pain scale; QUIP-RS: Questionnaire for Impulsive Compulsive Disorders in PD rating scale; ESS: Epworth Sleepiness Scale; PDSS-2: PD Sleep Scale version 2; NMSS: Non-motor symptoms scale; PFS: PD Fatigue Scale; AES: Apathy Evalutation Scale; PD-CRS: PD cognitive rating scale; MoCA: Montreal Cognitive Assessment.
