# Supplementary material for: Effects of safinamide on non-motor, cognitive, and behavioral symptoms in fluctuating Parkinson’s disease patients: a prospective longitudinal study
Source: Neurol Sci. 2021 May 24;43(1):357–64. doi: 10.1007/s10072-021-05324-w (PMC8724100; doi:10.1007/s10072-021-05324-w)
Supplement: Supplementary file 3 — (DOCX 26 kb). [file 10072_2021_5324_MOESM3_ESM.docx]

**Table S2.** Baseline and follow-up clinical features of PD patients switched from rasagiline to safinamide compared to iMAO-B naïve patients

| **Variables** | **iMAO-B naïve PD** | | **iMAO-B not naïve PD** | | ***P***  **iMAO-B naïve PD**  **vs**  **iMAO-B not naïve PD**  **at baseline** | ***P***  **iMAO-B naïve PD**  **vs**  **iMAO-B not naïve PD**  **at follow-up** | ***P***  ***for linear trend***  **iMAO-B naïve PD** | ***P***  ***for linear trend***  **iMAO-B not naïve PD** | ***P***  **iMAO-B naïve PD**  **vs**  **iMAO-B not naïve PD**  **Longitudinal changes** |
| --- | --- | --- | --- | --- | --- | --- | --- | --- | --- |
|  | **Baseline** | **6-month**  **follow-up** | **Baseline** | **6-month**  **follow-up** |  |  |  |  |  |
| **N** | **9** | | **11** | |  |  |  |  |  |
| **Age (years)** | 63.8±14.3 | - | 63.8±5.9 | - | N.S. | - | - | - | - |
| **Gender (M/W)** | 5/4 | - | 6/5 | - | N.S. | - | - | - | - |
| **UPDRS III** | 23.1±7.1 | 23.2±8.0 | 25.4±10.2 | 23.7±10.7 | N.S. | N.S. | N.S. | N.S. | N.S. |
| **Total LEDD (mg)** | 663.9±258.9 | 583.1±165.1 | 621.8±200.8 | 585.9±165.5 | N.S. | N.S. | N.S. | N.S. | N.S. |
| **LEDD Dopa (mg)** | 566.7±223.6 | 538.9±167.3 | 490.9±162.5 | 542.3±127.5 | N.S. | N.S. | N.S. | N.S. | N.S. |
| **LEDD DA (mg)** | 97.2±147.5 | 44.4±90.4 | 40.0±69.3 | 43.6±74.7 | N.S. | N.S. | N.S. | N.S. | N.S. |
| **UPDRS IV** | 5.0±2.5 | 4.3±2.0 | 2.8±2.3 | 3.4±2.3 | 0.05 | N.S. | N.S. | N.S. | N.S. |
| **AIMS** | 0.9±2.7 | 1.6±3.4 | 0.9±2.1 | 1.5±2.7 | N.S. | N.S. | N.S. | N.S. | N.S. |
| **NMSS total** | 33.1±19.0 | 28.6±27.8 | 78.0±59.9 | 37.4±21.3 | 0.037 | N.S. | N.S. | N.S. | N.S. |
| **NMSS Item 2.3** | 0.5±0.8 | 0.4±0.7 | 5.0±4.8 | 1.5±3.0 | 0.015 | N.S. | N.S. | N.S. | N.S. |
| **NMSS Item 2.4** | 2.5±3.9 | 0.3±1.0 | 5.7±4.7 | 4.5±3.9 | N.S. | 0.006 | N.S. | N.S. | 0.006 |
| **NMSS Item 3.8** | 1.0±3.0 | 0.0±0.0 | 5.6±4.6 | 1.4±2.2 | 0.015 | N.S. | N.S. | 0.009 | N.S. |
| **NMSS Item 3.9** | 0.7±1.4 | 1.3±4.0 | 4.9±5.0 | 1.8±3.1 | 0.020 | N.S. | N.S. | N.S. | N.S. |
| **BDI** | 3.8±2.5 | 3.2±4.4 | 9.4±5.2 | 9.5±4.6 | 0.008 | 0.013 | N.S. | N.S. | 0.013 |
| **PAS** | 6.4±5.9 | 6.1±3.9 | 16.5±7.9 | 7.6±4.6 | 0.005 | 0.018 | N.S. | N.S. | N.S. |
| **KPP** | 10.1±9.9 | 7.2±6.2 | 8.8±6.1 | 9.7±11.2 | N.S. | N.S. | N.S. | N.S. | N.S. |
| **QUIP-RS** | 1.0±2.1 | 0.7±1.6 | 0.1±0.3 | 1.0±1.8 | N.S. | N.S. | N.S. | N.S. | N.S. |
| **ESS** | 3.2±2.7 | 2.9±2.0 | 6.5±3.5 | 5.3±3.2 | 0.033 | N.S. | N.S. | N.S. | N.S. |
| **PDSS-2** | 125.3±14.1 | 119.1±21.1 | 110.5±24.6 | 123.4±15.0 | N.S. | N.S. | N.S. | N.S. | N.S. |
| **PFS** | 2.2±1.0 | 1.8±1.1 | 3.2±0.7 | 3.2±1.2 | 0.020 | N.S. | N.S. | N.S. | 0.018 |
| **AES** | 30.2±6.5 | 28.1±7.4 | 38.3±6.2 | 32.2±8.1 | 0.011 | N.S. | N.S. | 0.03 | N.S. |
| **MoCA** | 21.8±2.2 | 22.1±3.5 | 22.0±4.0 | 21.7±3.9 | N.S. | N.S. | N.S. | N.S. | N.S. |
| **PD-CRS (tot)** | 83.0±9.1 | 87.8±10.7 | 84.3±15.2 | 88.1±17.3 | N.S. | N.S. | N.S. | N.S. | N.S. |

Values are reported as mean ± standard deviation. Baseline and follow-up scores were compared by means of *t*-tests or Chi-squared tests as appropriate. For linear trend analysis, *P* values refer to repeated measures ANOVA models. One-way ANOVA was used to explore the differences in longitudinal changes of each clinical variable [(score *i*_follow-up_ – mean score *i*_baseline_) / Standard Deviation score *i*_baseline_] between the two PD subgroups. Abbreviations: iMAO-B: monoamine oxidase B inhibitors; PD: Parkinson’s disease; UPDRS: Unified Parkinson’s Disease Rating Scale: AIMS: Abnormal Involuntary Movements Scale; LEDD: Levodopa Equivalent Daily Dose; DA: dopamine agonist; BDI: Beck Depression Inventory; PAS: PD Anxiety Scale; KPP: King’s PD Pain scale; QUIP-RS: Questionnaire for Impulsive Compulsive Disorders in PD rating scale; ESS: Epworth Sleepiness Scale; PDSS-2: PD Sleep Scale version 2; NMSS: Non-motor symptoms scale; PFS: PD Fatigue Scale; AES: Apathy Evalutation Scale; PD-CRS: PD cognitive rating scale; MoCA: Montreal Cognitive Assessment.
