# Supplementary material for: Effects of safinamide on non-motor, cognitive, and behavioral symptoms in fluctuating Parkinson’s disease patients: a prospective longitudinal study
Source: Neurol Sci. 2021 May 24;43(1):357–64. doi: 10.1007/s10072-021-05324-w (PMC8724100; doi:10.1007/s10072-021-05324-w)
Supplement: Supplementary file 4 — (DOC 52 kb). [file 10072_2021_5324_MOESM4_ESM.doc]

**Supplementary references**

e1. Gibb WR, Lees AJ (1988) A comparison of clinical and pathological features of young- and old-onset Parkinson's disease. Neurology 38:1402-1406.

e2. Emre M, Aarsland D, Brown R, Burn DJ, Duyckaerts C, Mizuno Y, Broe GA, Cummings J, Dickson DW, Gauthier S, Goldman J, Goetz C, Korczyn A, Lees A, Levy R, Litvan I, McKeith I, Olanow W, Poewe W, Quinn N, Sampaio C, Tolosa E, Dubois B (2007) Clinical diagnostic criteria for dementia associated with Parkinson's disease. Mov. Disord. 22:1689-1707.

e3. Santangelo G, Siciliano M, Pedone R, Vitale C, Falco F, Bisogno R, Siano P, Barone P, Grossi D, Santangelo F, Trojano L (2015) Normative data for the Montreal Cognitive Assessment in an Italian population sample. Neurol. Sci. 36:585-591.

e4. Hoehn MM, Yahr MD (1967) Parkinsonism: onset, progression and mortality. Neurology 17:427-442.

e5. Fahn S, Elton RL (1987) UPDRS Development Committee. The Unified Parkinson’s Disease Rating Scale. In: Fahn S, Marsden CD, Calne DB, Goldstein M, editors, Recent developments in Parkinson’s Disease. 2nd edn Macmillan Healthcare Information; Florham Park, NJ

e6. Stacy M, Hauser R (2007) Development of a Patient Questionnaire to facilitate recognition of motor and non-motor wearing-off in Parkinson's disease. J. Neural. Transm. (Vienna) 114:211-217.

e7. Guy W (1976) Abnormal Involuntary Movement Scale. ECDEU assessment manual for psychopharmacology, Washington, DC: US Government Printing Office

e8. Tomlinson CL, Stowe R, Patel S, Rick C, Gray R, Clarke CE (2010) Systematic review of levodopa dose equivalency reporting in Parkinson's disease. Mov. Disord. 25:2649-2653.

e9. Chaudhuri KR, Martinez-Martin P, Brown RG, Sethi K, Stocchi F, Odin P, Ondo W, Abe K, Macphee G, Macmahon D, Barone P, Rabey M, Forbes A, Breen K, Tluk S, Naidu Y, Olanow W, Williams AJ, Thomas S, Rye D, Tsuboi Y, Hand A, Schapira AH (2007) The metric properties of a novel non-motor symptoms scale for Parkinson's disease: Results from an international pilot study. Mov. Disord. 22:1901-1911.

e10. Jenkinson C, Fitzpatrick R, Peto V, Greenhall R, Hyman N (1997) The Parkinson's Disease Questionnaire (PDQ-39): development and validation of a Parkinson's disease summary index score. Age Ageing 26:353-357.

e11. Nasreddine ZS, Phillips NA, Bédirian V, Charbonneau S, Whitehead V, Collin I, Cummings JL, Chertkow H (2005) The Montreal Cognitive Assessment, MoCA: a brief screening tool for mild cognitive impairment. J. Am. Geriatr. Soc. 53:695-699. Erratum in: J. Am. Geriatr. Soc. 67 (2019) 1991.

e12. Pagonabarraga J, Kulisevsky J, Llebaria G, García-Sánchez C, Pascual-Sedano B, Gironell A (2008) Parkinson's disease-cognitive rating scale: a new cognitive scale specific for Parkinson's disease. Mov. Disord. 23:998-1005.

e13. Santangelo G, Lagravinese G, Battini V, Chiorri C, Siciliano M, Abbruzzese G, Vitale C, Barone P (2017) The Parkinson's Disease-Cognitive Rating Scale (PD-CRS): normative values from 268 healthy Italian individuals. Neurol. Sci. 38:845-853.

e14. Beck AT, Ward CH, Mendelson M, Mock J, Erbaugh J (1961) An inventory for measuring depression. Arch. Gen. Psychiatry 4:561-571.

e15. Leentjens AF, Dujardin K, Pontone GM, Starkstein SE, Weintraub D, Martinez-Martin P (2014) The Parkinson Anxiety Scale (PAS): development and validation of a new anxiety scale. Mov. Disord. 29:1035-1343.

e16. Visser M, Marinus J, Stiggelbout AM, Van Hilten JJ (2004) Assessment of autonomic dysfunction in Parkinson's disease: the SCOPA-AUT. Mov. Disord. 19:1306-1312.

e17. Johns MW (1991) A new method for measuring daytime sleepiness: The Epworth Sleepiness Scale. Sleep: Journal of Sleep Research & Sleep Medicine 14:540-545.

e18. Trenkwalder C, Kohnen R, Högl B, Metta V, Sixel-Döring F, Frauscher B, Hülsmann J, Martinez-Martin P, Chaudhuri KR (2011) Parkinson's disease sleep scale--validation of the revised version PDSS-2. Mov. Disord. 26:644-652.

e19. Weintraub D, Mamikonyan E, Papay K, Shea JA, Xie SX, Siderowf A (2012) Questionnaire for Impulsive-Compulsive Disorders in Parkinson's Disease-Rating Scale. Mov. Disord. 27:242-247.

e20. Chaudhuri KR, Rizos A, Trenkwalder C, Rascol O, Pal S, Martino D, Carroll C, Paviour D, Falup-Pecurariu C, Kessel B, Silverdale M, Todorova A, Sauerbier A, Odin P, Antonini A, Martinez-Martin P; EUROPAR and the IPMDS Non Motor PD Study Group (2015) King's Parkinson's disease pain scale, the first scale for pain in PD: An international validation. Mov. Disord. 30:1623-1631.

e21. Marin RS, Biedrzycki RC, Firinciogullari S (1991) Reliability and validity of the Apathy Evaluation Scale. Psychiatry Res. 38:143–162.

e22. Brown RG, Dittner A, Findley L, Wessely SC (2005) The Parkinson fatigue scale. Parkinsonism Relat Disord, 11:49-55.
